# Development and Validation of a Semi-Analytical Predictive Model for Furniture Cabinet Performance from Corner Joints with Auxetic Fasteners

**DOI:** 10.3390/ma19122448

**Published:** 2026-06-08

**Authors:** Ersan Güray, Ali Kasal, Engin Ergin, Mehmet Yüksel, Harun Diler, Tolga Kuşkun

**Affiliations:** 1 Department of Civil Engineering, Faculty of Engineering, Muğla Sıtkı Koçman University, 48000 Muğla, Türkiye; ersan.guray@mu.edu.tr; 2 Department of Wood Products Industrial Engineering, Faculty of Technology, Muğla Sıtkı Koçman University, 48000 Muğla, Türkiye; enginergin@posta.mu.edu.tr (E.E.); myuksel@mu.edu.tr (M.Y.); tolgakuskun@mu.edu.tr (T.K.); 3 Department of Interior and Environmental Design, Faculty of Fine Arts, Afyon Kocatepe University, 03200 Afyonkarahisar, Türkiye; hdiler@aku.edu.tr

**Keywords:** auxetic fasteners, corner joints, furniture cabinet, moment capacity, stiffness

## Abstract

This study presents findings of an experimental and analytical approach investigating the relationship between the performance of individual corner joints and overall performance of furniture cabinets constructed with auxetic fasteners. The moment capacities and stiffness of cabinets assembled with different auxetic fasteners were also compared. For this purpose, 12 different auxetic fasteners were produced using three-dimensional printing technology. Cabinet bodies were constructed out of 18 mm particleboard (PB), while fasteners were produced using polylactic acid (PLA), acrylonitrile butadiene styrene (ABS), and acrylonitrile styrene acrylate (ASA) filaments. Cabinets were subjected to diagonal static loading to determine their moment capacity and stiffness. In total, 60 full-scale cabinets were prepared and tested using 12 different fasteners (3 filaments, 2 fastener lengths, and 2 fastener types), with 5 replications for each configuration. The results indicate that filament, and particularly fastener type, had a significant effect on moment capacity and stiffness, whereas fastener length was not statistically significant. Among the tested filaments, PLA exhibited the best performance. Cabinets assembled with H-type fasteners showed higher moment capacities and stiffness compared to those with K-type fasteners. The proposed semi-analytical model developed provides reasonable estimates for predicting the overall performance of cabinets based on individual corner joint tests.

## 1. Introduction

Currently, the panel furniture manufacturing industry generally uses mechanical fasteners (eccentric connectors such as Minifix, Multifix, screws, pullers, etc.) that do not require glue and allow for demountable assembly due to reasons such as ease of storage, transportation, and assembly. However, the costs of these fasteners and the technical and economic difficulties experienced in the assembly process are encouraging the design, production, and use of new alternative fasteners. In engineering and manufacturing, traditional materials are generally used in the production of mechanical fasteners. However, the interest in and need for smart materials is increasing every day, encouraging the design, production, and use of new alternative materials. Smart material designs and production are carried out by developing a new product or by adding extra features to traditional materials. In this context, within the scope of this study, pre-designed and optimized, invisible, innovative auxetic fasteners produced using 3D printing technology were used in the corner joints of panel-type furniture (chest of drawers, bedside tables, bookcases, kitchen cabinets, etc.). The design and optimization of these fasteners were previously carried out and validated in our earlier study using an artificial intelligence-based optimization approach (e.g., Artificial Bee Colony algorithm), and the resulting geometry was directly adopted in this work without further modification [1,2]. Auxetic fasteners are much easier to assemble than traditional fasteners and can be assembled manually with low mounting forces and without the need for any tools. In addition, the fasteners utilized in this study are demountable, similar to widely used eccentric connectors (e.g., Minifix and Multifix) and screws.

The Poisson ratio is one of the most important properties used to determine the structural behavior of materials used in many engineering fields today. While most materials used in engineering have a positive Poisson ratio, a very limited number of materials have a negative Poisson ratio, and these materials are called auxetic materials [3,4,5]. Auxetic materials and structures exhibit a range of advantageous mechanical behaviors, including improved shear and indentation resistance, synclastic deformation, tunable permeability, and enhanced energy absorption. Owing to these properties, they are considered suitable for applications such as protective equipment, cushioning systems, and vibration- or sound-damping materials. In addition, auxetic systems generally demonstrate superior hardness and toughness compared to conventional structures [6,7,8,9]. However, these improvements are typically more evident when comparisons are made on a similar mass or density basis.

Interest in auxetic materials is increasing, and consequently, scientific studies on these materials are becoming more widespread. These materials are attracting attention, especially as an alternative to traditional materials, and the fact that the Poisson ratio, which has a very fundamental effect on mechanical behavior, is negative in auxetic materials, unlike traditional materials, provides these materials with a superior property, encouraging research into their use in engineering applications. A negative Poisson ratio is important for furniture assembly, as it causes the fastener to expand when subjected to tensile and/or bending forces during use, resulting in a stronger joint; conversely, it causes the fastener to contract when subjected to compressive forces during assembly, reducing the mounting forces [10]. There are a limited number of studies on the application of auxetic materials in the furniture industry. In summary, studies in this field have focused on developing different auxetic helical spring designs with negative Poisson ratios to improve comfort and durability in upholstery of seating furniture used in homes and offices. These designs have been numerically modeled and tested under long-term fatigue and static loads in laboratory conditions. As a result, it has been reported that auxetic helical spring designs can be used as an alternative to traditional upholstery materials in seating furniture upholstery systems, thus providing more comfortable and durable upholstery systems [11,12,13]. In another study, Smardzewski 2013 [14] developed lightweight wood panel materials with a cellular structure and auxetic properties for panel furniture and tested their mechanical properties. In another study based on modeling and experimentation, lightweight honeycomb textured wood sandwich composite (mixture of wood powder and polylactic acid) panels with auxetic properties were developed, and their elastic properties were determined [15]. Furthermore, the mounting forces and dowel withdrawal tests of some auxetic dowel designs developed for use in furniture assembly and the moment capacity and stiffness of L-shaped corner joints connected with auxetic dowels were examined. The mounting forces, withdrawal strength, moment capacity and stiffness of the reference dowels and auxetic dowels were compared experimentally and numerically within the scope of the studies [16,17,18]. It was reported that auxetic nails do not always have an advantage over non-auxetic nails, that small deformations in auxetic nails can be predicted in a virtual environment with finite element models in a manner consistent with real deformations, and that the limitations in auxetic nail design were determined thanks to the experimental and numerical results obtained [19].

Regarding cabinet furniture joints, previous studies have extensively investigated the structural behavior of corner joints and the performance of various connection systems under various loading conditions. Experimental studies on corner joints have shown that single-dowel connections exhibit higher moment resistance under tension than compression and that increasing the dowel diameter and length generally improves joint strength [20]. For multi-dowel configurations, an optimal spacing of around 75 mm was reported to maximize the moment capacity per connector [21]. In addition, Liu and Eckelman 1998 [22] demonstrated that joint strength increases with fastener interaction up to the point where influence zones begin to overlap, after which the improvement becomes limited. Krzyżaniak et al. 2021 [23] examined the effect of additively manufactured connectors on the moment capacity and stiffness of corner joints. Their results indicate that connector geometry and internal stress distribution play a critical role in joint performance and that 3D-printed connection elements can significantly enhance or reduce structural resistance depending on their design parameters. In a comparative study, Janíková et al. 2024 [24] evaluated different furniture corner joint systems, including cam fittings, dowels, and hybrid connections. The findings reveal that certain hybrid configurations can more than double the moment capacity compared to conventional joint systems, highlighting the strong influence of joint type on overall structural behavior. From a numerical perspective, Ban and Choi 2022 [25] analyzed the deformation behavior of different corner joint types under bending loads using computational methods. Their study demonstrated that joint stiffness is a governing factor in the global stability of furniture structures and directly affects deformation response under service loads. In the literature, a number of studies have also focused on the structural performance of full cabinet systems. In one study, a finite element-based model was developed to analyze stress and strain distribution in furniture corner joints by representing fastening components as experimentally determined elastic elements, enabling accurate prediction of joint behavior and overall cabinet deflection, with results validated against the existing literature [26]. In studies carried out by Yüksel et al. 2014 [27] and Kasal et al. 2011 [28] the performance of both four-sided (without back panel) and five-sided (with back panel) panel furniture in real dimensions under static load was investigated. In a study by Ho and Eckelman 1994 [29] a cyclic stepped increasing loading model was used in five-sided cabinet tests. Another study demonstrates that CAE-based furniture stiffness modeling can be improved by introducing a joint elasticity modulus, enabling more realistic numerical simulations of L-type joints and providing a validated framework for comparing and selecting optimal joint solutions in cabinet furniture design [30]. One study emphasizes that the structural performance of storage furniture is strongly dependent on corner joint type and geometry and that determining the actual load demand at joints is essential for selecting and designing an adequate number of safe and appropriate connections [31]. In one study, an approach was proposed to estimate the front to back loading capacity of a chair frame from individual joint tests [32]. Studies in the literature show that the resistance of a structural frame system to static loads is approximately twice its resistance to cyclic fatigue loads [33,34,35].

The literature review indicates that previous studies have examined the mechanical performance of corner joints as well as the structural behavior of four-sided and five-sided cabinet systems in panel furniture. However, these studies have generally addressed these aspects independently, without establishing a direct and systematic relationship between individual joint performance and the overall cabinet response. This lack of a predictive or integrative approach represents a key limitation in the existing literature. In this context, the present study contributes to the field by experimentally and analytically linking corner joint performance to the global behavior of furniture cabinets, thereby addressing this gap.

This study is a continuation of a previous study [1,2] in which individual L-shaped corner joint tests were performed and aims to experimentally and analytically investigate the part–whole relationships in the performance of furniture cabinets. Briefly, this study focuses on developing a theoretical model using inductive methods to estimate the overall strength of five-sided furniture cabinets from the data obtained from the individual joint tests. Accordingly, the hypothesis of the study is as follows: “In five-sided cabinet furniture, there is a relationship between the individual performance of corner joints and the performance of the system as a whole. Hence, the strength performance of five-sided furniture cabinets can be reasonably predicted thanks to the data obtained from the individual corner joint tests”.

## 2. Materials and Methods

### 2.1. Wood-Based Panels and Filaments

In this study, an 18 mm-thick melamine-coated PB (Kastamonu Entegre, Kayseri, Türkiye) was used for the cabinet bodies, and a 4 mm-thick melamine-coated medium-density fiberboard (MDF) (Kastamonu Entegre, Kayseri, Türkiye) was used for the back panels. The wood-based panels were obtained randomly from a commercial company in the market. Moisture content (MC) values and densities (d) of the PB and MDF were determined in accordance with ASTM D 4442 (2020) and ASTM D 1037 (2013), respectively [36,37]. Average MC values were 7.64 and 6.72%, and d values were 0.65 and 0.73 g/cm^3^ for PB and MDF, respectively. In addition, the mechanical properties of the PB, including bending strength (MOR), modulus of elasticity (MOE), and internal bonding (IB) strength, were determined in accordance with ASTM D1037 (2013) [37], while the shear modulus (G) was determined according to Eckelman (2003) [33]. The obtained values were 14.93 MPa for MOR, 4264 MPa for MOE, 0.47 MPa for IB strength, and 1565 MPa for G.

Polylactic acid (PLA) filament (Porima, İstanbul, Türkiye) was chosen for the production of auxetic fasteners due to its bio-based and biodegradable nature, while acrylonitrile butadiene styrene (ABS) filament (Porima, İstanbul, Türkiye) and acrylonitrile styrene acrylate (ASA) filaments (Porima, İstanbul, Türkiye) were preferred because, although they are petroleum-based and non-biodegradable, they exhibit high mechanical strength and are widely used engineering thermoplastics with relatively low toxicity and limited direct environmental or human health impacts during their service life. The densities of the PLA, ABS, and ASA filaments, as well as their tensile strength and modulus of elasticity values, were determined according to the principles specified in the ASTM D3039/D3039M–17 standards [38]. All test specimens used for the determination of these properties were manufactured using a 3D printer under identical printing conditions to ensure consistency and comparability of the results. The densities of the PLA, ABS, and ASA filaments were measured as 1.24, 1.04, and 1.05 g/cm^3^, respectively. The tension strengths of the filaments were 50, 36, and 39 MPa, while the modulus of elasticities were 937, 747, and 797 MPa, respectively, for PLA, ABS, and ASA.

### 2.2. Production of the Fasteners and Assembling the Furniture Cabinets

**Figure 1:**
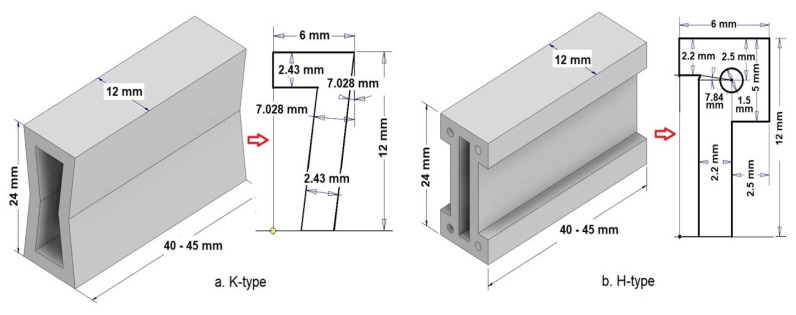
K–type (**a**) and H–type (**b**) fasteners with their quarter cross-sections [1,2].

In this study, auxetic fasteners, previously designed and optimized [1,2] as an alternative to mechanical fasteners, such as eccentric connectors (Minifix, Multifix, screws, etc.), were utilized in corner joints in the production of five-sided furniture cabinets (Figure 1). The design of the fasteners aimed to provide a connection for panel furniture corner joints that is easy to assemble and disassemble and does not require any tools during assembly.

The fasteners were designed in two different lengths, 40 mm and 45 mm. The cross-sectional outer dimensions of the fasteners were 24 × 12 mm. 

**Figure 2:**
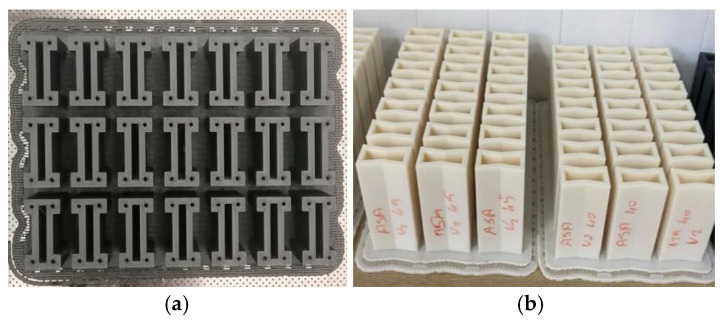
Produced H-type (**a**) and K-type (**b**) auxetic fasteners with 3D printing technology.

Auxetic fasteners were produced using 3D printing technology. The printing was carried out using a Zortrax 3D printer (Zortrax, Poland; Türkiye distributor), and the infill pattern was defined as grid. All specimens were manufactured with a 90% infill ratio. For the production of fasteners, three different filaments, PLA, ABS, and ASA, which are the most commonly used in 3D printers, were utilized. Production conditions were determined separately for each filament based on company recommendations and preliminary tests [1,2]. For PLA, the nozzle temperature was set to 220 °C and the bed temperature to 70 °C; for ABS, the nozzle temperature was set to 240 °C and the bed temperature to 90 °C; and for ASA, the nozzle temperature was set to 260 °C and the bed temperature to 90 °C. Standard Zortrax Z-Suite slicing parameters (3.x series slicing software) were used with a nozzle diameter of 0.4 mm and a layer height of 0.14 mm; other slicing settings, including perimeters, infill strategy, and print speed, followed the default manufacturer profile. The printing orientation was selected such that the fasteners were built perpendicular to the printer bed, resulting in the layer-stacking direction being aligned with the loading direction, as shown in Figure 2, with the cavity surfaces facing upward. In this configuration, the applied loads during the test did not act directly in the weakest interlayer (layer adhesion) direction, thereby reducing the risk of shear failure between layers.

**Figure 3:**
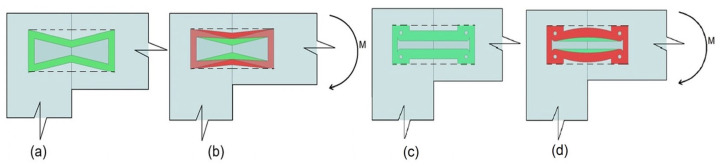
Auxetic fasteners before loading (green) (**a**,**c**) and possible deformation after loading (red) (**b**,**d**).

The expected typical mechanical behavior of auxetic fasteners used in the corner joints of furniture cabinets under moment is shown in Figure 3.

In corner joints, due to the loads subjected during use, the fasteners in the joint (Figure 3a,c) undergo an expanding deformation, as illustrated schematically in Figure 3b,d, putting more pressure on the side walls of the groove. This, combined with increased friction, makes it more difficult for the fastener inside the groove to dislocate under load. In other words, the initial form of the fasteners, indicated in Figure 3a,c, undertakes the deformations indicated in Figure 3b,d after loading, resulting in a stronger and more durable furniture joint. It should be noted that the deformed geometry shown in Figure 3b,d are schematic representation intended to illustrate the deformation mechanism and does not correspond to an actual modification of the groove geometry. The fastener remains confined within the predefined groove, and the interaction occurs through contact with the groove side walls. Here, the increase in pressure on the inner surface of the groove due to the moment occurs through the unusual deformation of the fastener thanks to the negative Poisson ratio.

**Figure 4:**
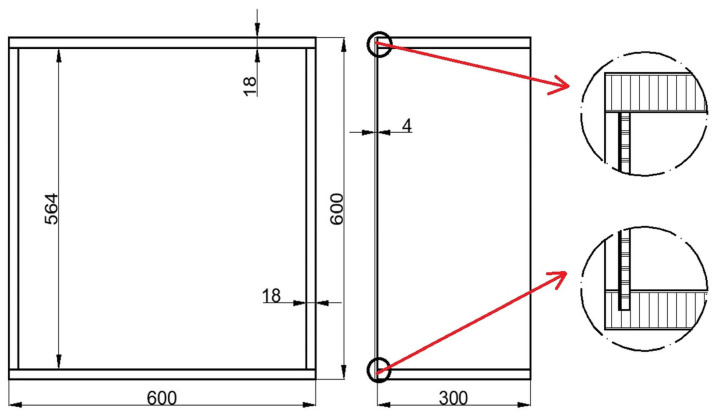
The dimensions of the test cabinets utilized in this study.

Cabinets were manufactured with dimensions of 600 mm × 600 mm × 300 mm. Each cabinet consists of 5 members: 2 horizontal PB members (bottom and top panels), measuring 600 mm × 300 mm × 18 mm; 2 vertical members (side panels), measuring 564 mm × 300 mm × 18 mm; and a 4 mm back panel (Figure 4).

**Figure 5:**
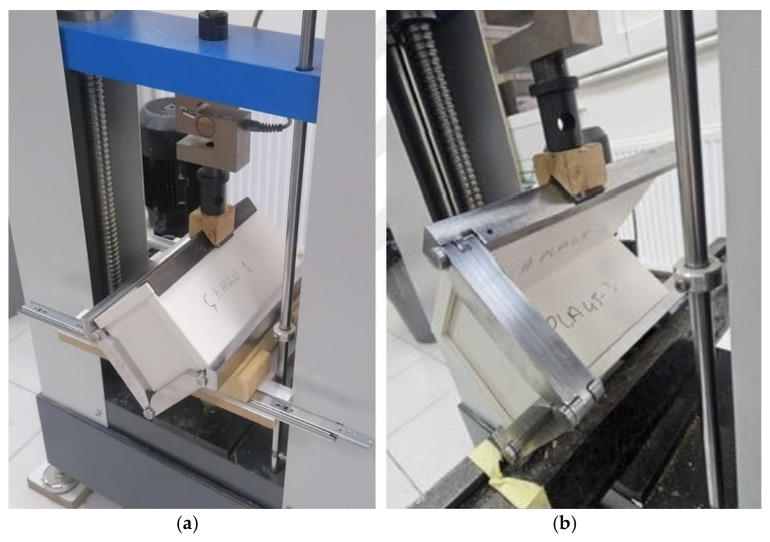
Diagonal compression (**a**) and tension (**b**) tests of individual corner joints [1,2].

The test cabinet was designed with a back panel to reflect real-life furniture usage conditions, as commercial storage furniture is typically used with a back panel installed. The L-type corner joint tests were previously conducted in the earlier study [1,2] where the specimens were also prepared and tested with a back panel configuration using a specially designed test apparatus to ensure consistency with real cabinet boundary conditions (Figure 5).

The same joint configuration was adopted in the present work for the full cabinet specimens to ensure consistency and enable a reliable correlation between individual corner joint and full cabinet system behavior.

All edges of the cabinet panels, except for the back panel, were covered with 2 mm PVC edge bands. It should be considered that PVC edge banding may have positively affected the pull-out behavior of the fasteners due to changes in edge material characteristics.

At the corner joints, the horizontal and vertical members were connected to each other with 2 fasteners; therefore, 8 fasteners were used in each cabinet. Firstly, grooves with the appropriate geometry and dimensions for the type of fastener were cut using a CNC machine (Biesse, Pesaro, Italy) at the locations where the fasteners were to be placed. The grooves were machined into the edges of the vertical members (side panels) and into the mating surfaces of the horizontal members (bottom and top panels). The groove width and depth were defined as 24 mm and 12 mm, respectively. The groove length was determined according to the fastener length, resulting in total groove lengths of 80 mm and 90 mm for 40 mm and 45 mm-length fasteners, respectively. The grooves were positioned at a distance of 35 mm and 25 mm from the front of the panel edge for 40 mm and 45 mm-length fasteners, respectively, and 70 mm from the back of the panel edge for both lengths consistent with the fastener placement. The distances between the two grooves were 55 mm and 45 mm, while the distances between the assembled two fasteners were 95 mm and 90 mm for the 40 mm and 45 mm-length fasteners, respectively.

**Figure 6:**
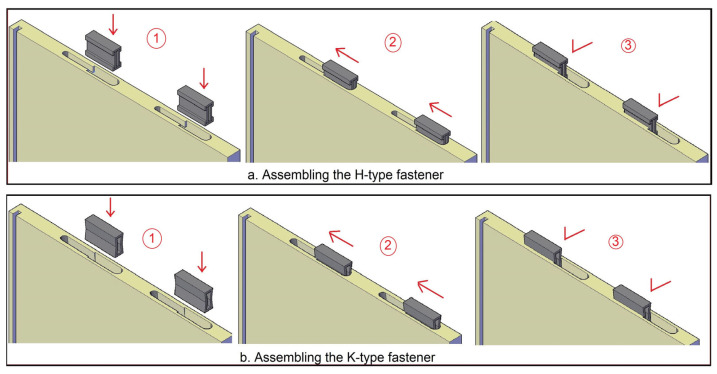
Inserting method of the H-type (**a**) and K-type (**b**) fasteners into the grooves.

The groove length was designed as twice the fastener length. One half of the groove was machined to precisely match half of the fastener cross-sectional geometry in order to ensure a tight fit using a custom-fabricated cutting tool designed specifically for each fastener geometry, while the remaining half was machined as a round-end rectangular clearance zone which is designed to match the outer cross-sectional dimensions of the fastener using a cylindrical cutting tool to allow for initial insertion and subsequent lateral sliding of the fastener into its final position. The fastener was first inserted into the initial groove and then slid laterally into the main groove, which was designed to closely match the fastener geometry and ensure a tight fit (Figure 6).

The connection between the fasteners and the grooves was designed as a tight-fit (interference-fit) assembly, and all cabinets were manufactured and assembled under controlled conditions to ensure consistent fitting and proper load transfer across all tests.

**Figure 7:**
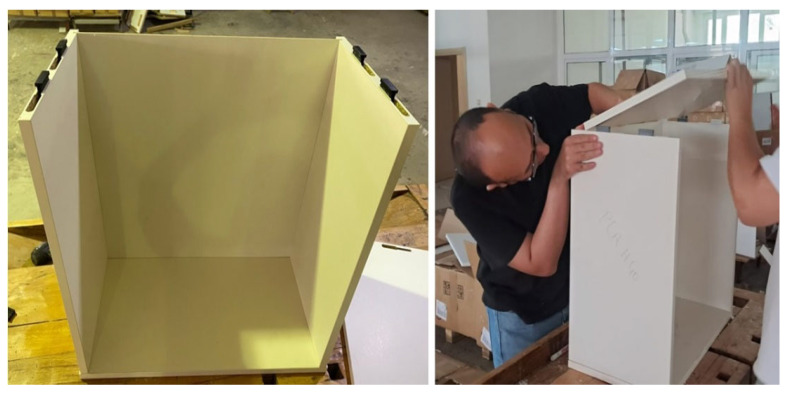
Assembly process of the test cabinets.

During assembly, the bottom panel and the side panels were first joined by inserting the fasteners. The fastener was first inserted into an initial groove matching its outer dimensions and then slid laterally into a geometry-specific groove to achieve a tight fit. For the assembly sequence, fasteners were positioned by hand into the grooves on the surfaces of the bottom panel and then engaged with the grooves on the edges of the side panels through a sliding motion, allowing for a tool-free assembly with low mounting forces. After this stage, the back panel was installed by sliding it into pre-cut grooves located only on the bottom and the two side panels, which had been assembled in the previous step. The grooves were designed with a nominal width of 5 mm, while the back panel thickness was 4 mm, providing an approximately 1 mm clearance to facilitate assembly. The groove depth was 9 mm, corresponding to half of the thickness of the cabinet body panels. Finally, the top panel was assembled using fasteners, completing the cabinet construction. The back panel was not inserted into any groove on the top panel; instead, it remained in direct contact with the top panel surface (Figure 7).

All test cabinets were kept at 20 °C ± 2 °C and 65% ± 3% relative humidity until their weight became stable (approximately 1 month) before testing in order to reach an equilibrium moisture content (MC).

### 2.3. Static Diagonal Testing of the Furniture Cabinets

In the tests, a diagonal load was applied to the mid-depth of the corner of test cabinets. An L-shaped metal component was used to ensure uniform distribution of the applied load along the depth direction of the cabinet. The loading type forced all the joints in the same way as the forces subjected by the joints in the individual diagonal compression and tension tests of corner joints [1,2]. Static loading tests were carried out at a speed of 6 mm/min on a computer-controlled 50 kN-capacity universal testing machine (Mares, İstanbul, Türkiye). The loading rate was selected based on previous studies [28,39,40] to ensure quasi-static loading conditions.

The performance of the test cabinets was taken as the moments (*M_test_*) carried by the joints under the test loads. In addition, the vertical displacements (*y*) occurring under the test loads were determined, and the stiffness (*K_test_*) of the joints were calculated according to the elastic (linear relationship) region of the moment–rotation graphs plotted accordingly. Based on commonly accepted approaches in the literature, the elastic region was assumed to correspond to the range between 10% and 40% of the maximum load [18,23,30,41]. Within this interval, a linear regression analysis was applied to the moment–rotation data, and the slope of the fitted line was taken as the stiffness value. The goodness of fit of the regression was verified by the coefficient of determination (R^2^), ensuring that the selected range exhibited a strong linear relationship. The same loading range was consistently used for all test groups to ensure comparability of the calculated stiffness values.

**Figure 8:**
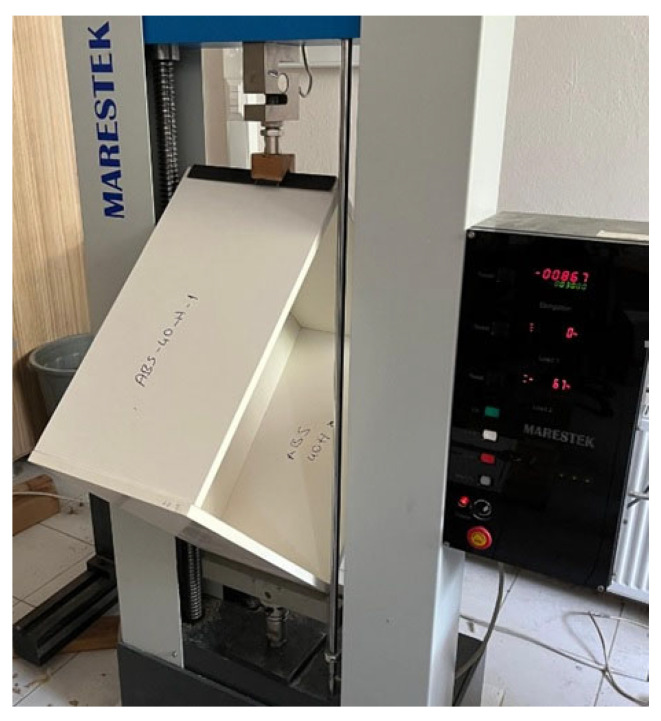
Static diagonal testing of the test cabinets.

It should be noted that the load application at the mid-depth of the cabinet resulted in a non-uniform load distribution between the front and rear regions due to the presence of the back panel. As a consequence, the rear side experienced reduced deformation compared to the front side where higher displacement was observed. In this study, displacement measurements were taken at the mid-span under the loading point to represent the global structural response of the system, and local displacement differences were not separately evaluated. Accordingly, the measured stiffness reflects the combined structural response of the corner joints, rear panel, and overall cabinet geometry under the applied loading conditions. The test setup and load application method are shown in Figure 8.

### 2.4. Experimental Design and Statistical Evaluation

Altogether, 12 sets of test cabinets consisting of 5 replications, or a total of 60 five-sided cabinets, were prepared for tests. The specimen schedule is shown in Table 1.

Full linear models (Model 1 and Model 2) for the three-way factorial experiments were considered to evaluate the influence of filament material (PLA, ABS, and ASA), fastener length (40 and 45 mm) and fastener type (auxetic pattern) (K-type and H-type) on the moment capacity and stiffness of furniture cabinets under static loads. The form models are as follows:

*M_ijkl_* = *μ* + *A_i_* + *B_j_* + *C_k_* + *(A* × *B)_ij_* + *(A* × *C)_ik_* + *(B* × *C)_jk_* + *(A* × *B × C)_ijk_* + *ρ_l_* + *ε_ijkl_* (1)

*K_ijkl_* = *μ* + *A_i_* + *B_j_* + *C_k_* + *(A* × *B)_ij_* + *(A* × *C)_ik_* + *(B* × *C)_jk_* + *(A* × *B* × *C)_ijk_* + *ρ_l_* + *ε_ijkl_* (2)

where *M_ijkl_* = moment capacity (Nm); *K_ijkl_* = stiffness (Nm/rad); *μ*_1_ = population mean moment capacity for all combinations (Nm); *μ*_2_ = population mean stiffness for all combinations (Nm/rad); *A* = a discrete variable representing the effect of filament material; *B* = a discrete variable representing the effect of fastener length; *C* = a discrete variable representing the effect of fastener type; (*A* × *B*), (*A* × *C*), and (*B* × *C*) = effects of the two-way interactions among the three variables; (*A* × *B* × *C*) = the effect of the three-way interactions among the three variables; *ρ* = the effect of the replication; *ε* = a random error term; *i* = an index for filament material, 1…3; *j* = an index for fastener length, 1…2; *k* = an index for fastener type, 1…2; and *l* = an index for the replication, 1…5.

Three factor analyses of variances (ANOVA) as a general linear model procedure were separately performed for individual data for moment capacities and stiffness values to analyze main effects and their two-way and three-way interactions on the moment capacities and stiffness values. Then, a least significant difference (LSD) multiple comparison procedure at a 5% significance level was performed to determine the mean differences of moment capacities and stiffness values in static diagonal loading considering the significant main effects and two-factor and three-factor interactions in the ANOVA results. The Minitab (2023) statistical software was utilized for the statistical analyses in this study.

### 2.5. A Semi-Analytical Model for Predicting the Performance of the Overall Cabinet from the Individual Corner Joint Tests

**Figure 9:**
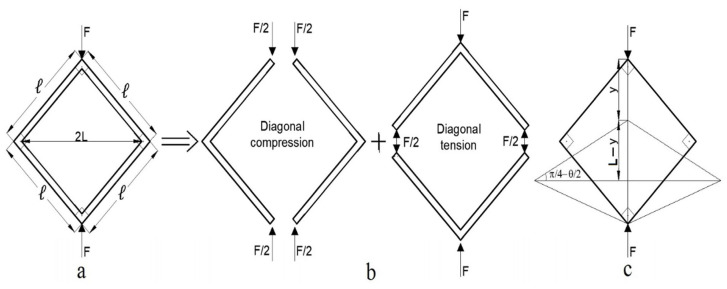
Loading form (**a**), simultaneous diagonal compression and tension (**b**), and typical deformation (**c**) during the cabinet testing.

In the developed semi-analytical model, the performance of the test cabinets under static diagonal loading (Figure 9a) is considered as a linear combination of the moment and stiffness values obtained from diagonal compression and diagonal tensile tests in the previous study [1,2]. Since diagonal loading creates two separate types of loading that are independent of each other and do not affect each other in the cabinet joints (Figure 9b), the results produced by these loadings are added linearly together in the applied methodology.

For any type of fastener, the moment capacity ($M_{test}$) of the test cabinet was calculated using the maximum force values obtained from the experiments, according to Equation (3):

(3) $$M_{test}\;=\;F\times L\;\;\;\;(\mathrm{Nm})$$

Here, F is the maximum force obtained from the tests (N), and L is the moment arm (m). The moment arm was calculated using Equation (4), since the cabinet dimensions are 600 mm × 600 mm (l = 0.6 m). Accordingly, the moment arm was obtained as 0.4242 m.

(4) $$L=l\;\times \;\frac{\sqrt{2}}{2}\;\;\;\;(m)$$

The stiffness of the cabinets was first calculated using the displacement (y) values obtained from the tests and the angular deformation (θ) (Figure 9c) corresponding to the $M_{test}$ values in the elastic region (Equation (5)).

(5) $$\theta =2\left[\arccos\;\left(\frac{\sqrt{2}}{2}-\frac{y}{l}\right)-\frac{\pi }{4}\right]\;\;\;\;(\mathrm{rad})$$

The angular deformation was calculated based on a rigid frame assumption, which is valid within the small deformation range considered in this study. Accordingly, the stiffness coefficients ($K_{test}$), which indicate the rigidity of the test cabinet, were calculated using Equation (6):

(6) $$K_{test}\;=\;\frac{M_{test}}{\theta }\;\;\;\;(\mathrm{Nm}/\mathrm{rad})$$

For any type of fastener, when the moment capacity and stiffness values under diagonal compression are defined as $M_{C}$ and $K_{C}$, and the moment capacity and stiffness values under diagonal tension are defined as $M_{T}$ and $K_{T}$, obtained from the individual joint tests; the moment capacity (${Mh}_{theo}$, ${Mk}_{theo}$) and stiffness (${Kh}_{theo}$, ${Kk}_{theo}$) of the test cabinets, connected with H-type and K-type auxetic fasteners, respectively, are theoretically expressed as linear combinations by Equations (7)–(10).


(7) $${Mh}_{theo}={\;c}_{h}\times 2\left(M_{C}+M_{T}\right)\;\;\;\;(\mathrm{Nm})$$

(8) $${Mk}_{theo}={c\;}_{k}\times 2\left(M_{C}+M_{T}\right)\;\;\;\;(\mathrm{Nm})$$

(9) $${Kh}_{theo}={\;d}_{h}\times 2\left(K_{C}+K_{T}\right)\;\;\;\;(\mathrm{Nm}/\mathrm{rad})$$

(10) $${Kk}_{theo}={\;d}_{k}\times 2\left(K_{C}+K_{T}\right)\;\;\;\;(\mathrm{Nm}/\mathrm{rad})$$

Here, $c_{h}$ and $c_{k}$ are the constant coefficients determined for the moment capacity of the H-type and K-type auxetic fasteners, respectively, while $d_{h}$ and $d_{k}$ are the constant coefficients determined for stiffness. In the developed model, separate coefficients were determined for the H-type and K-type fasteners as a result of numerous preliminary analyses, and it was assumed that the moment capacity and stiffness values obtained from individual diagonal compression and diagonal tension tests contributed equally to the moment capacity and stiffness of the test cabinets. Then, the moment capacity and stiffness values obtained from the cabinet tests were subjected to regression analysis using the least squares method. According to the analysis results, $c_{h}$, $c_{k}$, $d_{h}$, and $d_{k}$, which can most closely predict the moment capacity and stiffness values of the test cabinets from the moment capacity and stiffness values of the H-type and K-type individual joint test, regardless of filament material and fastener length factors, were obtained as follows (Equations (11)–(14)).

(11) $$\sum_{i=1}^{N}R_{i}=\sum_{i=1}^{N}{\left({\left\{M_{test}-{2c}_{h}\left(M_{C}+M_{T}\right)\right\}}_{i}\right)}^{2}$$

(12) $$\sum_{i=1}^{N}R_{i}=\sum_{i=1}^{N}{\left({\left\{M_{test}-2c_{k}\left(M_{C}+M_{T}\right)\right\}}_{i}\right)}^{2}$$

(13) $$\sum_{i=1}^{N}R_{i}=\sum_{i=1}^{N}{\left({\left\{{\;K}_{test}-{2d}_{h}\left(K_{C}+K_{T}\right)\right\}}_{i}\right)}^{2}$$

(14) $$\sum_{i=1}^{N}R_{i}=\sum_{i=1}^{N}{\left({\left\{K_{test}-{2d}_{k}\left(K_{C}+K_{T}\right)\right\}}_{i}\right)}^{2}$$

Here, $R_{i}$ is the residual for each type of fastener, and *N* is the number of fastener types. The appropriate $c_{h}$, $c_{k}$, $d_{h}$, and $d_{k}$ values for minimizing this sum are determined by the extremum of the residual functions as given in the following expressions (Equations (15)–(18)).

(15) $$\frac{d}{dc_{h}}\;\sum_{i=1}^{N}R_{i}=\sum_{i=1}^{N}2\left({\left\{M_{test}-{2c}_{h}\left(M_{C}+M_{T}\right)\right\}}_{i}\right)\cdot \left(-2{\left\{M_{C}+M_{T}\right\}}_{i}\right)=0$$

(16) $$\frac{d}{dc_{k}}\;\sum_{i=1}^{N}R_{i}=\sum_{i=1}^{N}2\left({\left\{M_{test}-2c_{k}\left(M_{C}+M_{T}\right)\right\}}_{i}\right)\cdot \left(-2{\left\{M_{C}+M_{T}\right\}}_{i}\right)=0$$

(17) $$\frac{d}{dd_{h}}\;\sum_{i=1}^{N}R_{i}=\sum_{i=1}^{N}2\left({\left\{{\;K}_{test}-{2d}_{h}\left(K_{C}+K_{T}\right)\right\}}_{i}\right)\cdot \left(-2{\left\{K_{C}+K_{T}\right\}}_{i}\right)=0$$

(18) $$\frac{d}{dd_{k}}\;\sum_{i=1}^{N}R_{i}=\sum_{i=1}^{N}2\left({\left\{K_{test}-{2d}_{k}\left(K_{C}+K_{T}\right)\right\}}_{i}\right)\cdot \left(-2{\left\{K_{C}+K_{T}\right\}}_{i}\right)=0$$

Using the coefficients obtained with this semi-analytical approach, the theoretical moment capacity and stiffness values for each test cabinet group were calculated and compared with the moment capacity and stiffness values obtained from actual static performance tests.

## 3. Results and Discussion

In this study, statistical evaluations were carried out regarding the moment capacities and stiffness values of the cabinets subjected to performance tests with static diagonal loading. In the following sections, failure modes of the test cabinets and results of the significance and mean comparison analyses of the moment capacity and stiffness data are presented, and the semi-analytical model developed to predict the overall cabinet performance from joint tests is described. Finally, the actual test results of the moment capacity and stiffness were compared to the theoretical results obtained from the semi-analytical predictive model.

### 3.1. Failure Modes Observed from the Tests

All test cabinets showed similar failure modes in terms of general deformation in the experiments. With the application of the diagonal test force, the test cabinets underwent vertical displacement from the corner where the force was applied. As a result, the corner joints where the force was applied and the opposite support were located were forced to opening (the angle increased), while the corner joints on the sides were forced to closing (the angle decreased). Accordingly, it can be said that two of the four corner joints in the test cabinets were under diagonal tension, and the other two were under diagonal compression. This failure pattern is consistent with the typical deformation pattern obtained from individual corner joint tests under diagonal compression and tension.

**Figure 10:**
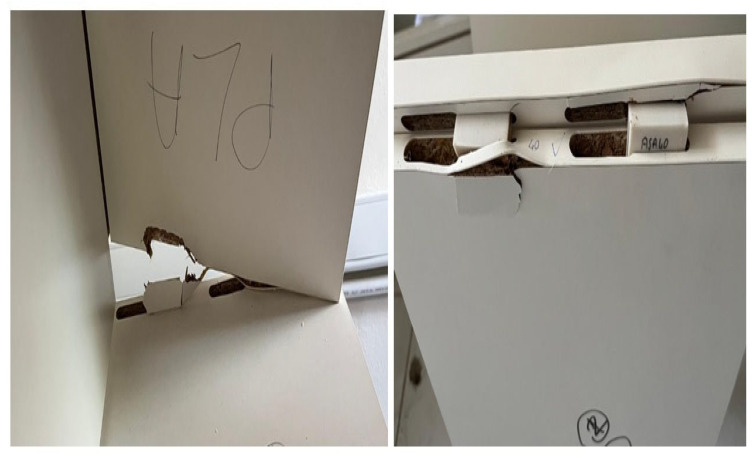
Failure modes of the corner joints under static diagonal force, showing damage in the arm opening (tension-dominated) corners.

The experimental process was not accompanied by any fracture or visible damage in the auxetic fasteners. Post-test inspections confirmed that no permanent deformation or interlayer cracking occurred in the fasteners. The observed failure modes are shown in Figure 10.

The observed failure modes in the corner joints were primarily governed by the particleboard material, where the fasteners were extracted from their grooves by localized crushing and splitting of the board perpendicular to the surface due to variations in joint angles (increase or decrease). The failure mode presented in Figure 10 corresponds to the opening region.

### 3.2. Moment Capacity and Stiffness Results of the Test Cabinets

Table 2 shows the ANOVA results for the effects of filament, fastener type and fastener length on moment capacities and stiffness of test cabinets under diagonal static loading.

According to the results of the analysis of variance (ANOVA), the main effects of filament material and fastener type, as well as the three-way interaction effect, were found to be statistically significant at the 0.05 significance level. However, the effects of fastener length and all other two-way interactions on moment capacity were found to be insignificant at the 0.05 significance level. Although fastener length does not exhibit a significant main effect, the significant interaction suggests that its influence depends on the combined effects of filament type and fastener type rather than acting independently. When the calculated F-values in Table 2 are examined, it is possible to conclude that the effect of fastener type (i.e., the auxetic pattern) on moment capacity of cabinets is greater, while the filament is less effective.

In the case of stiffness, a similar trend was observed. Filament type and fastener type had statistically significant effects (*p* < 0.05), whereas fastener length did not show a significant influence. Among the interactions, only the filament×fastener type interaction was found to be significant, while the other two-way interactions and the three-way interaction were not statistically significant. Based on the F-values, fastener type was again identified as the most dominant factor affecting stiffness, consistent with the findings for moment capacity.

Table 3 compares the effects of the filament material and fastener type main factors on the moment capacities and stiffness values of the cabinets.

When the results are examined, it is clear that there are not very big differences among the filament materials used in fastener production. However, mean comparisons based on the filament material revealed that the cabinets assembled with the PLA fasteners yielded the highest moment capacity values. There were no statistically significant differences in moment capacities between the cabinets assembled with fasteners made from ABS and ASA materials. The cabinets assembled with the fasteners produced from ASA were found to have lower moment capacities. Cabinets assembled with the fasteners made from PLA showed an average of a 2.91% higher moment capacity compared to those made from ABS and a 5.48% higher moment capacity compared to those made from ASA. In this context, PLA material could be recommended because it is both environmentally friendly and biodegradable, and also cheaper than the other filaments.

Regarding the mean comparisons based on fastener type, the cabinets assembled with H-type fasteners yielded significantly higher moment capacity values than those assembled with K-type fasteners. The moment capacity values obtained from cabinets assembled with H-type connectors were found to be 80% higher than the moment capacity value obtained from the cabinets assembled with K-type connectors. Accordingly, the H-type fastener, among the fastener types tested in this study, could be recommended for cabinet assembly.

Similarly, for stiffness, PLA-based fasteners yielded the highest values, while ABS and ASA exhibited lower and partially overlapping groupings. In terms of fastener type, the H-type fasteners provided significantly higher stiffness values compared to the K-type ones.

Overall, the results obtained for moment capacity and stiffness are consistent with each other. In both cases, PLA showed the best performance among the filament materials, and the cabinets connected with the H-type fastener demonstrated superior performance compared to the cabinets connected with the K-type one.

Table 4 compares the effects of the “filament×fastener type×fastener length” three-way interaction on the moment capacities of the test cabinets. Since the three-way interaction for stiffness values was found to be statistically insignificant, homogeneity groups are not provided for stiffness in this table; only mean values and coefficients of variation are presented.

The results presented in Table 4 indicate that the highest moment capacity values were obtained with the cabinets assembled with the 40 mm-length H-type ABS fasteners (1000.40 Nm). In contrast, the lowest moment capacity values were observed with the cabinets assembled with the 40 mm-length K-type ABS fasteners (505.61 Nm). In general, the H-type fasteners exhibited significantly higher moment values compared to the K-type fasteners across all material types. This finding suggests that fastener type (auxetic pattern) has a dominant influence on moment capacity. Conversely, K-type fasteners consistently resulted in lower moment capacities for all tested materials. Regarding fastener length, the 45 mm configuration generally provided higher moment capacity values compared to 40 mm. However, this trend was not consistent across all material–fastener combinations. For PLA, the H-type fastener results for 40 mm and 45 mm fall within overlapping homogeneity groups, indicating no statistically significant difference. A similar behavior was observed for ASA with H-type fasteners, where partial overlap between the groups suggests no clear statistical separation. For ABS, although a numerical decrease was observed for H-type fasteners at 45 mm, the difference was not sufficiently consistent to establish a uniform trend across all materials. According to the mean comparison results for the three-way interaction from a material perspective, ABS generally achieved the highest moment capacity values, followed by PLA and ASA. Nevertheless, in some cases, particularly for the H-type fastener, these differences are not statistically significant due to overlapping group classifications.

Table 5 compares the effects of the “filament*fastener type” two-way interaction on the stiffness values of the test cabinets.

The results in Table 5 indicate that the highest stiffness values were obtained from the H-type PLA fasteners (30,658.5 Nm/rad), which formed a distinct group. This was followed by the H-type ABS fasteners (28300.0 Nm/rad) and H-type ASA fasteners (28,632.1 Nm/rad), which were statistically similar and placed in the same homogeneity group. Among the K-type fasteners, ABS (21,863.6 Nm/rad) showed relatively higher stiffness compared to the other K-type combinations and formed a separate group, whereas the PLA fasteners (20,733.8 Nm/rad) and ASA fasteners (20,708.3 Nm/rad) exhibited the lowest stiffness values and were not significantly different from each other. It is evident that the H-type fasteners consistently provided higher stiffness values regardless of filament type, while the K-type fasteners resulted in considerably lower stiffness.

Overall, it can be concluded that fastener type is the most significant parameter affecting mechanical performance properties such as moment capacity and stiffness, with the H-type fastener providing superior performance. Fastener length, on the other hand, exhibits a secondary and limited effect on the overall mechanical behavior.

### 3.3. Performance Prediction Results of the Test Cabinets from the Individual Joint Tests

A simple calibrated model has been developed to predict the moment capacities ($M_{test}$) and stiffness ($K_{test}$) of test cabinets joined with H-type and K-type fasteners based on the experimental moment capacity ($M_{C}$, $M_{T}$) and stiffness values ($K_{C}$, $K_{T}$) obtained from diagonal compression and tension tests of L-type corner joint specimens prepared with the same fastener types and dimensions. Subsequently, the theoretical moment capacity ($M_{theo}$) and stiffness ($K_{theo}$) values calculated for the test cabinets according to this semi-analytical approach were compared with the moment capacity and stiffness values obtained for the test cabinets from actual tests.

The moment capacity and stiffness values obtained from diagonal compression and tension tests of L-type individual corner joint specimens connected with each type of fasteners are given in Table 6. These results were taken from the preliminary study of this research [1,2].

As a result of the calculations and analyses, the coefficients for moment capacity were found to be 2.58 ($c_{h}$ = 2.58) for the H-type fastener and 1.62 ($c_{k}$ = 1.62) for the K-type fastener, while in case of the stiffness, the coefficients were found to be 5.00 ($d_{h}$ = 5.00) for the H-type fastener and 3.89 ($d_{k}$ = 3.89) for the K-type fastener. The reliability of the developed model was evaluated using the coefficient of determination (R^2^) and standard deviations. The obtained R^2^ values were 0.91, 0.90, 0.88, and 0.87, while the corresponding standard deviations were 0.22, 0.16, 0.58, and 0.50 for $c_{h}$, $c_{k}$, $d_{h}$ and $d_{h}$, respectively, indicating good agreement between the experimental and predicted results. Accordingly, the moment capacity and stiffness values obtained for the L-type corner joints as a result of diagonal compression and tension tests in Table 6 were substituted into the related equations (Equations (7)–(10)) for each type of fastener, and the theoretical moment (${Mh}_{theo}$, ${Mk}_{theo}$) and stiffness (${Kh}_{theo}$, ${Kk}_{theo}$) values of the test cabinets for the relevant fastener were obtained. The stiffness coefficients in the predictive model represent system-level parameters that include the combined structural contributions of the corner joints, back panel connection, and overall cabinet geometry. The model is formulated based on cabinet configurations with identical back-panel assemblies and boundary conditions, and its applicability is limited to similar structural configurations.

Following this stage, the moment capacity ($M_{test}$) and stiffness ($K_{test}$) values calculated for the whole cabinet system from the actual tests of the cabinets were compared with the theoretically calculated moment capacity and stiffness values, and the reliability of the developed semi-analytical predictive approach was examined. The comparison results regarding the moment capacity and stiffness values calculated according to the data obtained from the actual tests of the cabinets and the theoretical moment capacity and stiffness values estimated according to the developed model are given in Table 7 with differences.

According to Table 7, the theoretical moment capacity values calculated using the semi-analytical predictive model developed in this study showed a high degree of agreement with the experimental moment capacity data calculated using the maximum force values obtained from the experiments. The fact that the differences between the test and theoretical moment values were very low for all fastener types, except for the largest difference of 20% (ABS × K-type × 40 mm), demonstrates the reliability of the model developed in this study. The “Test/Theoretical” moment capacity ratios ranged from 0.86 to 1.20 for all groups; it can be clearly seen that the model converged highly with the experimental data in the moment capacity predictions. The fact that the ratios were very close to 1 supports the accuracy of the model. In particular, for the PLA and ABS filaments, the differences were kept below 9% for the H-type fastener test cabinets. The moment capacity values for the PLA × K-type × 40 mm (0%), ABS × H-type × 40 mm (1%), ABS × K-type × 45 mm (1%), and ASA × K-type × 40 mm (2%) groups were almost perfectly predicted.

According to Table 7, the calculated theoretical stiffness values also showed consistent results with the experimental stiffness data calculated using the vertical displacement values obtained from the experiments. The fact that the differences between test and theoretical stiffness values were very low for all fastener types, except for the largest difference of 24% (ABS × K-type × 40 mm), demonstrates the reliability of the semi-analytical model developed in this study. The “Test/Theoretical” stiffness ratios ranged from 0.85 to 1.24 for all groups, and it was observed that the model approached the experimental data to a high degree in stiffness predictions. The fact that the ratios were very close to 1 supports the accuracy of the model for stiffness as well. Particularly in the ASA filaments, the differences between the experimental and theoretical stiffness values in cabinets with both the H-type and K-type fasteners were found to be 5% or less.

These results demonstrate that the semi-analytical approach used in this study provides a valid and reasonable model for estimating the moment capacity and stiffness of the entire system using experimental data obtained from individual joint tests. 

## 4. Conclusions

This study investigates the effects of filament material, auxetic pattern, and fastener length on the moment capacity and stiffness of furniture cabinets assembled with auxetic fasteners. Furthermore, a semi-analytical approach has been developed to predict the overall structural performance of the cabinet using the experimental performance data obtained from the individual corner joint tests.

The results confirm that fastener type has a substantial influence on moment capacity and stiffness, with the H-type fastener generally providing higher performance compared to the K-type one across all materials. This indicates that auxetic pattern may play a key role in determining structural behavior, while the K-type fastener shows limited effectiveness. The influence of fastener length appears to be limited, as the increase from 40 mm to 45 mm did not consistently result in statistically significant improvements. While ABS showed relatively higher moment values, the differences among the filament materials were less pronounced when used with the H-type fastener. Overall, optimizing the auxetic pattern is more critical than modifying the fastener length or filament selection.

The overall moment capacity and stiffness of the whole cabinet system were successfully predicted through a semi-analytical model developed based on the experimental data obtained from the individual joint tests. In other words, the innovative aspect of the study is the development of a unique, holistic model that reliably predicts the structural performance of the entire system using the data obtained from small-scale joint tests. The coefficients obtained for the equations (Equations (7)–(10)) in this study (moment: 2.58 for H-type and 1.62 for K-type; stiffness: 5.00 and 3.89, respectively) can be directly used as practical design parameters for engineering applications. The developed model makes it possible to predict the structural behavior of the entire system with only small-scale tests focusing on connection details, thus enabling engineering design without the need for large-scale prototype specimens. While traditional experimental approaches involve time-consuming and costly processes, especially due to the preparation and testing of large-scale prototypes, this method allows for the strength and rigidity properties of the entire system to be predicted with results obtained from only small and more easily applicable joint tests. This will significantly increase efficiency in R&D activities. In addition, the proposed model can be extended to different cabinet geometries and material configurations; however, the numerical coefficients are specific to the tested cabinets and fastener system and should be recalibrated when applied to different cabinets.

From a sectoral perspective, the benefits provided by this model are particularly important for companies operating in the furniture and fastener manufacturing industry. The furniture sector must optimize many parameters simultaneously, such as durability, ease of assembly, and aesthetics. The model developed here offers the possibility of quickly and economically testing the performance of different filament materials and fastener geometries, thus providing flexibility in design and production processes. This model reduces the number of prototypes produced and therefore costs, while also saving time in the product development process. Furthermore, the elimination of the need for large-scale sample production reduces the environmental impact of testing processes, contributing to sustainable production. In addition, obtaining reliable data on the overall strength of the system through small-scale joint tests allows companies to evaluate product performance in advance, optimize design parameters, and detect potential design flaws at an early stage. This increases product reliability and customer satisfaction, providing a competitive advantage. Moreover, the demonstration of the model’s validity on different material combinations and connection types makes it easily adaptable to various applications in the industry.

In conclusion, this study not only provides valuable contributions in terms of theoretical modeling and experimental validation in a scientific sense but also develops a model that can be directly integrated into practical engineering applications and industrial processes. The developed model is a significant tool for saving time and costs while improving product quality and design reliability. Particularly in the furniture industry, this method is critical for accelerating and optimizing production processes and has the potential for widespread adoption in industrial applications.

## 5. Patents

A national patent application (2024/3 BBF) entitled “H-type Auxetic Fastener for Panel Furniture” was filed on 20 September 2024 by the Technology Transfer Office Coordination of Muğla Sıtkı Koçman University, with Muğla Sıtkı Koçman University as the rights holder. The H-type fastener was originally developed within the scope of TÜBİTAK Project No. 122O887 (TÜBİTAK 1005—National New Ideas and New Products Research Funding Program). In this study, the proposed fastener was experimentally evaluated through full-scale cabinet tests. The patenting process is still ongoing.

## Figures and Tables

**Table 1:** Experimental design used in the study.

No	Filament	Fastener Type	Fastener Length	Replication
1	PLA	H-type	40 mm	5
2			45 mm	5
3		K-type	40 mm	5
4			45 mm	5
5	ABS	H-type	40 mm	5
6			45 mm	5
7		K-type	40 mm	5
8			45 mm	5
9	ASA	H-type	40 mm	5
10			45 mm	5
11		K-type	40 mm	5
12			45 mm	5
Total Number of Test Cabinets				60 Cabinets

**Table 2:** ANOVA results for moment capacities and stiffness of the cabinets.

	Sources	Degrees of Freedom	Sum of Squares	Mean Squares	F-Value	Significance (*p* < 0.05)
Moment Capacity (Nm)	Filament	2	16,254	8127	7.06	0.002
	Fastener Type	1	2,783,500	2,783,500	2418.21	0.000
	Fastener Length	1	286	286	0.25	0.620
	Filament × Fastener Type	2	4683	2342	2.03	0.142
	Filament × Fastener Length	2	2117	1059	0.92	0.406
	Fastener Type × Fastener Length	1	1435	1435	1.25	0.270
	Filament × Fastener Type × Fastener Length	2	8321	4160	3.61	0.035
	Error	48	55,251	1151		
	Total	59	2,871,847			
Stiffness (Nm/rad)	Filament	2	10,663,006	5,331,503	4.82	0.012
	Fastener Type	1	982,917,971	982,917,971	888.89	0.000
	Fastener Length	1	453,888	453,888	0.41	0.525
	Filament × Fastener Type	2	30,639,996	15,319,998	13.85	0.000
	Filament × Fastener Length	2	86,453	43,227	0.04	0.962
	Fastener Type × Fastener Length	1	19,044	19,044	0.02	0.896
	Filament × Fastener Type × Fastener Length	2	4,080,271	2,040,136	1.84	0.169
	Error	48	53,077,560	1,105,783		
	Total	59	1,081,938,189			

**Table 3:** Mean comparison of moment capacity and stiffness by filament material and fastener type.

Filament	Moment		Stiffness		Fastener Type	Moment		Stiffness	
	X (Nm)	(HG)	X (Nm/rad)	(HG)		X (Nm)	(HG)	X (Nm/rad)	(HG)
PLA	774.54	A	25,696.2	A	H-Type	969.19	A	29,196.9	A
ABS	752.59	B	25,081.8	AB					
ASA	734.28	B	24,670.2	B	K-Type	538.42	B	21,101.9	B

X: mean value; HG: homogeneity group.

**Table 4:** Mean comparison of moment capacities based on the three-way interaction and mean stiffness values of all cabinet groups.

Filament	Fastener Type	Fastener Length	Moment			Stiffness	
			X (Nm)	COV (%)	(HG)	X (Nm/rad)	COV (%)
PLA	H-type	40 mm	975.85	3.53	ABC	30,863.31	2.82
		45 mm	988.33	3.26	AB	30,453.66	2.42
	K-type	40 mm	552.43	2.28	DE	20,271.71	2.16
		45 mm	581.56	6.92	D	21,195.95	3.68
ABS	H-type	40 mm	1000.40	6.83	A	27,913.87	2.70
		45 mm	960.24	3.40	ABC	28,686.15	6.09
	K-type	40 mm	505.61	2.50	F	22,058.88	4.63
		45 mm	544.10	2.72	DEF	21,668.39	1.69
ASA	H-type	40 mm	939.44	4.70	C	28,499.01	7.02
		45 mm	950.88	2.01	BC	28,765.13	2.86
	K-type	40 mm	535.99	6.34	EF	20,767.71	5.21
		45 mm	510.81	2.78	EF	20,648.92	3.32

COV: coefficients of variation.

**Table 5:** Mean comparison of stiffness values based on the two-way interaction.

Filament	Fastener Type	Stiffness	
		X (Nm/rad)	(HG)
PLA	H-type	30,658.5	A
	K-type	20,733.8	D
ABS	H-type	28,300.0	B
	K-type	21,863.6	C
ASA	H-type	28,632.1	B
	K-type	20,708.3	D

**Table 6:** Moment and stiffness values of L-type individual corner joint elements [1,2].

Filament	Fastener Type	Fastener Length	Diagonal Compression		Diagonal Tension	
			Moment (*M_C_*) (Nm)	Stiffness (*K_C_*) (Nm/rad)	Moment (*M_T_*) (Nm)	Stiffness (*K_T_*) (Nm/rad)
PLA	H-type	40 mm	102.49	1977.02	75.18	650.96
		45 mm	114.51	2208.85	68.77	711.84
	K-type	40 mm	90.62	2422.08	79.48	512.99
		45 mm	79.47	2477.60	111.62	516.38
ABS	H-type	40 mm	119.41	2649.51	76.50	616.34
		45 mm	117.51	2527.97	54.12	707.79
	K-type	40 mm	56.62	1718.36	73.39	575.44
		45 mm	64.92	2341.01	104.98	602.79
ASA	H-type	40 mm	131.27	2317.20	81.68	519.48
		45 mm	122.41	2157.78	48.76	609.26
	K-type	40 mm	65.40	2011.65	103.01	502.72
		45 mm	72.20	2204.50	97.70	573.12

**Table 7:** Comparison of moment and stiffness values from the tests and theoretically calculated.

Filament	Fastener Type	Fastener Length	Moment (Nm)			Stiffness (Nm/rad)		
			Test (M_test_)	Theoretical (*M_theo_*)	Test/Theo.	Test (*K_test_*)	Theoretical (*K_theo_*)	Test/Theo.
PLA	H-type	40 mm	975.85	914.65	1.07	30,863.31	26,279.80	1.17
		45 mm	988.33	943.53	1.05	30,453.66	29,206.90	1.04
	K-type	40 mm	552.42	551.12	1.00	20,271.71	22,776.14	0.89
		45 mm	581.55	619.13	0.94	21,195.95	23,233.28	0.91
ABS	H-type	40 mm	1000.37	1008.54	0.99	27,913.87	32,658.50	0.85
		45 mm	960.24	883.55	1.09	28,686.15	32,357.60	0.89
	K-type	40 mm	505.61	421.23	1.20	22,058.88	17,799.89	1.24
		45 mm	544.10	550.48	0.99	21,668.39	22,843.89	0.95
ASA	H-type	40 mm	939.44	1096.27	0.86	28,499.01	28,366.80	1.00
		45 mm	950.88	881.18	1.08	28,765.13	27,670.40	1.04
	K-type	40 mm	535.99	545.65	0.98	20,767.71	19,511.51	1.05
		45 mm	510.81	550.48	0.93	20,648.92	21,554.33	0.96

## Data Availability

The data presented in this study are available from the corresponding author upon reasonable request.
